# Distinctive Roles of Sirtuins on Diabetes, Protective or Detrimental?

**DOI:** 10.3389/fendo.2018.00724

**Published:** 2018-11-29

**Authors:** Jie Song, Bing Yang, Xiaobin Jia, Mingyu Li, Wei Tan, Shitang Ma, Xinhong Shi, Liang Feng

**Affiliations:** ^1^School of Traditional Chinese Pharmacy, China Pharmaceutical University, Nanjing, China; ^2^Affiliated Hospital on Integration of Chinese and Western Medicine, Nanjing University of Chinese Medicine, Nanjing, China; ^3^Nanjing University of Chinese Medicine, Nanjing, China; ^4^Life and Health college, Anhui Science and Technology University, Fengyang, China

**Keywords:** SIRTs, diabetes, insulin resistance, glucose uptake, fatty acid oxidation

## Abstract

Dysregulation of metabolic pathways leads to type 2 diabetes, characteristic of high glucose concentration caused by insulin resistance. The histone deacetylases sirtuins exhibit remarkable enzymatic activities. Accumulating evidence indicates that sirtuins can be pharmacologically activated to ameliorate diabetes. Here, we evaluated different roles of sirtuins (SIRT1-SIRT7) in diabetes progression and described their involvement in metabolic pathways of skeletal muscle, adipose tissue and liver. The nuclear sirtuins, SIRT1, SIRT6, and SIRT7, regulate the activity of key transcription factors and cofactors in almost all tissues with the cellular responses to energy demands. The mitochondrial sirtuins, SIRT3, SIRT4, and SIRT5, regulate the activity of mitochondrial enzymes in response to fasting and calorie restriction. Moreover, genetic polymorphisms of SIRT1 and SIRT2 have been reported to associate with diabetes development. It's worth noting that SIRT1, SIRT2, SIRT3, and SIRT6 are positive regulators of insulin resistance in most cases. In the opposite, SIRT4 and SIRT7 inhibit insulin secretion and fatty acid oxidation. Identification of SIRT1 activators for diabetes has gained wide attention, such as metformin, resveratrol, and resveratrol derivatives. Randomized, prospective, and large-scale clinical trials are warrant to uncover the responsibilities of SIRTs modulators on diabetes progress.

## Introduction

**Figure 1:**
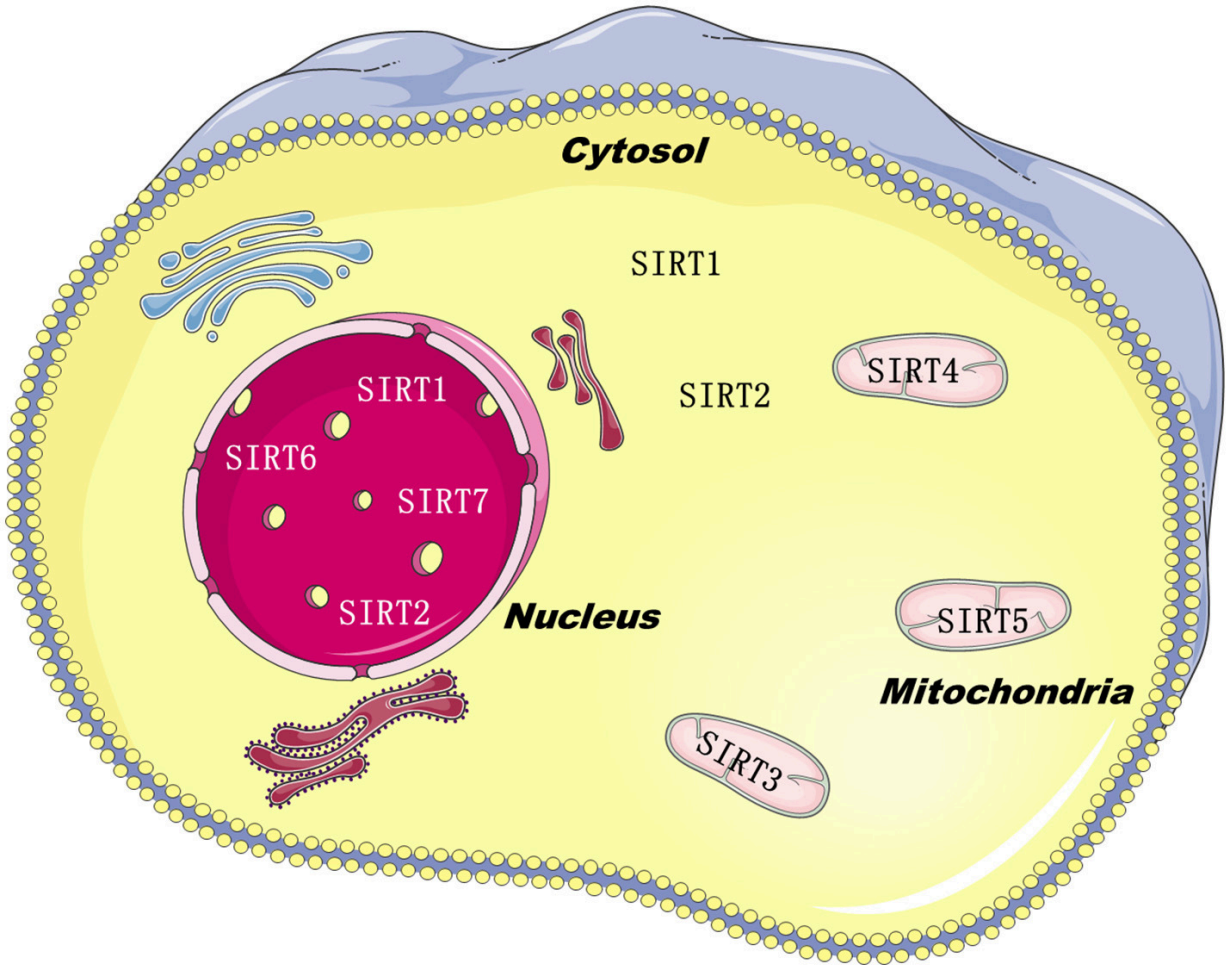
The localization of SIRTs family in the cells.

Sirtuins belong to class III histone deacylases, and in each deacylation cycle one molecule NAD^+^ is consumed (1). SIRTs isoforms have been defined in mammals, such as SIRT1–7. Although they are equipped with a highly conserved structure of about 275 amino acids, just like the silent information regulator 2 in yeast (2), the C-and N-terminal extensions are distinctive, which are the predominant factor of sirtuins subcellular localization (3). SIRT1, SIRT6, and SIRT7 are principally found in the nucleus. SIRT2 is mainly located in the cytoplasm, and SIRT3-5 are located in the mitochondria (Figure 1). The catalytic core is made up of a small zinc-binding domain, a large Rossmann-fold domain, and a few flexible loops which bind these domains together. The large domain of most sirtuins resembles each other, characteristic of a β-sheet encircled by six α-helices, excluding SIRT2, which has seven α-helices (4). In the small domain, diversities are observed in the principal sequence. Firstly, the helix bundle is only absent in SIRT7 and SIRT6. Next, SIRT5 and SIRT4 have a loop and a short helix, yielding an insertion in the small domain. This feature might be essential for the mitochondrial localization (5). Lastly, SIRT1 has a 5-residue loop in this domain, neighboring to the zinc-binding cysteine. Those dissimilarities in the catalytic core might closely relate with their key properties.

SIRT1-3 and SIRT6 exhibit remarkable demyristoylase activity (6, 7). Except for functioning as an ADP-ribosyltransferase (8, 9), SIRT4 also possesses a lipoamidase effect (10). By targeting carbamoyl phosphate synthetase (CPS1), SIRT5 can remove malonyl or succinyl groups in a manner very similar to deacetylation (11, 12). SIRT6 deacetylates histone H3K9 & H3K56, and mono-ADP-ribosyltransferate long-chain acyl and acetyl groups (13). SIRT7 is distributed in all organs and tissues (14) and activates RNA polymerase I transcription. Although several of its molecular substrate have been identified, including WSTF–ISWI chromatin remodeling complex (WICH), rDNA transcription factor UBF (the nucleolar upstream-binding factor) and RNA polI (15), SIRT7's catalytic activity remains elusive.

Diabetes is a global epidemic problem growing exponentially, posing a serious threat. Type 2 diabetes mellitus (T2DM) is a multifactorial metabolic and endocrine disorder for human beings, characteristic of abnormal glucose level in blood. T2DM individuals are estimated to be 642 million by 2,040 globally (16). T2DM is predominately attributed to insulin resistance and pancreatic β-cell dysfunction (17, 18). Insulin resistance, primarily in liver, muscle and adipose tissue as well, spoils glucose disposal, leading to β-cell insulin increase and hyperinsulinemia in a compensatory manner.

Blunted sirtuin activity has been reported to induce diabetes and metabolic syndrome, and aggravate high-fat diet (HFD) effects in mice. Exceptionally, SIRT4 prevents insulin secretion and stimulates T2DM. SIRT4 also negatively regulates fatty acid oxidation (FAO) in muscle and liver cells. A mutation in human SIRT1 caused a familial form of autoimmune diabetes (19–21). SIRT1 can interact with transcription factors and co-activators (RelA/p65, FOXO, and p53). T2DM group has lower SIRT1 mRNA levels compared with healthy group. There is a negative connection between fasting plasma glucose and SIRT1, as shown in the correlation analysis. The expression of SIRT1 in monocytes and granulocytes of T2DM might associate with glucose/lipid metabolism status (22). In both of the kidney and liver of diabetic rats, SIRT1 and SIRT2 gene expressions reduced considerably than blank control group (23). HFD triggers SIRT1 decrement in mice probably via proteolysis (19). SIRT1 expression is also reduced in obese humans (24, 25), and meanwhile diabetes is alleviated in SIRT1-overexpressed mice (26, 27, 28). SIRT6 is important for sustaining pancreatic β-cell function in mice. SIRT6 knockout leads to severe hypoglycemia in mice. SIRT6 deficiency results in liver steatosis and accelerates insulin resistance and obesity induced by diet. Overnutrition and aging decreased SIRT6 level as well as irregular lipid and glucose metabolism (29). SIRT7 deficiency in mice induces multi-systemic mitochondrial dysfunction. To fully understand the part SIRTs play in diabetes and to reveal regulatory mechanisms regarding SIRTs is the principal purpose in the current review.

## SIRTs in insulin resistance

Pancreatic β cells secret insulin after nutrient stimulation. In the fed state, glycolysis, glucose uptake, and glycogen formation will be promoted by insulin. The glucose homeostasis in adipose tissue and skeletal muscle can also be regulated by insulin (30). Insulin resistance will give rise to hyperuricemia, visceral adiposity, dyslipidemia, hypertension, and hyperglycemia. About 70% of glucose is disposed in muscle. Free fatty acids accumulation and inflammation in muscle triggeres abundant lipid deposition. Imbalanced muscle uptake promotes extra glucose to returns to the liver, which yields intense circulation of free fatty acids, finally leading to fat accumulation and insulin resistance.

**Figure 2:**
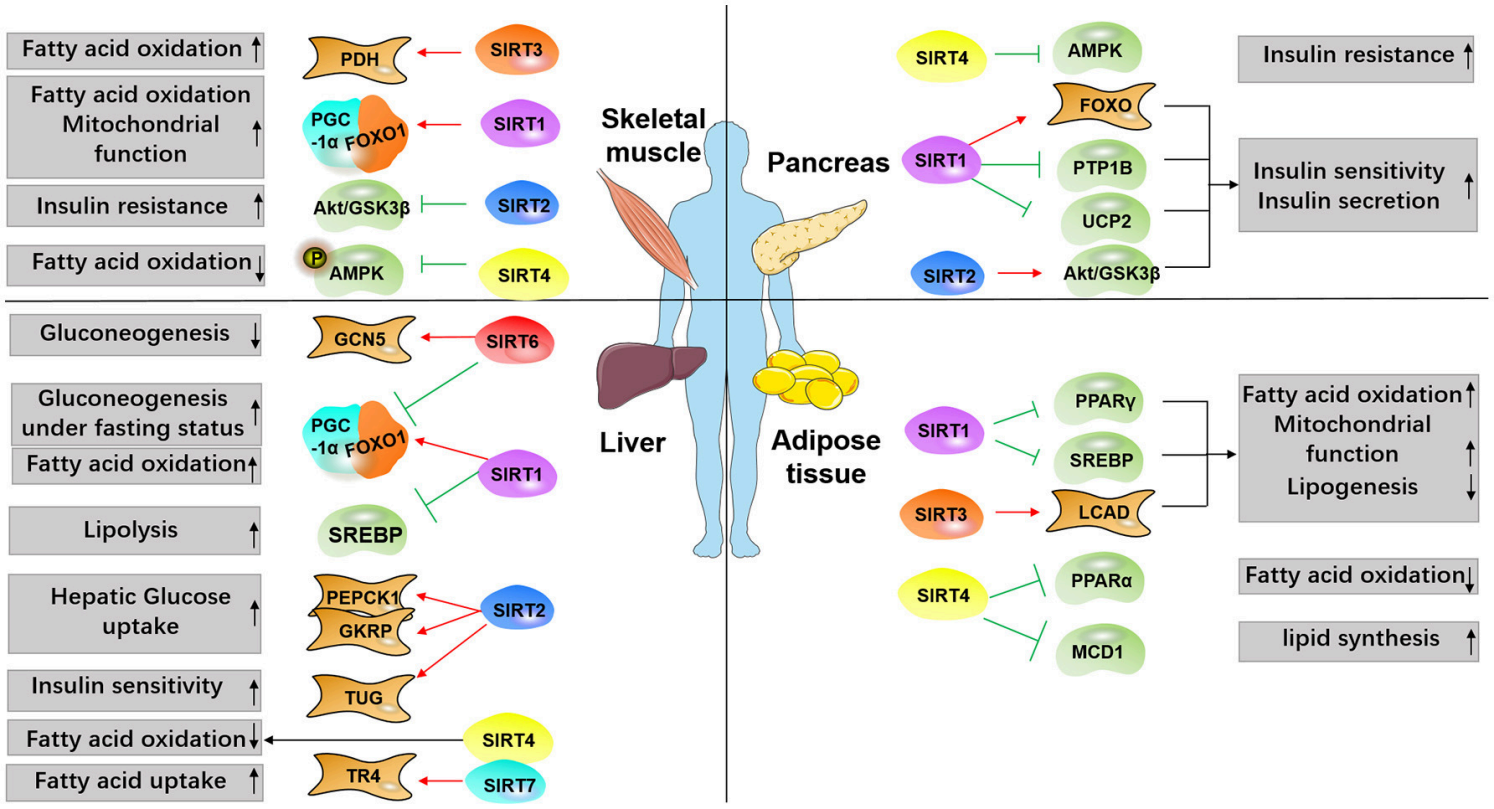
Overview of SIRTs targets involved in diabetes. Major metabolic tissues, including liver, pancreas, adipose, and skeletal muscle are depicted to demonstrate SIRTs functions in the metabolic process modulating insulin resistance, glucose uptake, and lipid synthesis. Red arrows indicate promoting effect, and green arrows indicate inhibiting effect.

As a major inhibitor of the insulin receptor, protein tyrosine phosphatase 1b (PTP1B) can be inhibited by SIRT1, thus increasing insulin sensitivity (31–33). In insulin-resistant obese mice, PTP1B level was raised, associated with decreased SIRT1 expression in skeletal muscle. SIRT1 overexpression brought PTP1B expression to the baseline and abrogated the insulin-stimulated signaling in skeletal muscle (Figure 2 and Table 1). In β-cells, SIRT1 overexpression boosted insulin secretion and improved glucose tolerance, contributing to glucose homeostasis (34). Uncoupling protein 2 (UCP2) disturbs the electrochemical proton gradient, leading to shrink in ATP production and insulin secretion impairment afterwards (35). SIRT1 overexpression suppressed UCP2, finally enlarging ATP levels and insulin secretion (34, 36). UCP2 expression in MIN6 β-cells was reduced by SIRT1, as demonstrated in microarray assays. SIRT1 RNAi decreased the secretion capability of β-cells, which was renovated by UCP2 RNAi (36). On the other side, oxidative stress-induced hyperglycemia and cytokine toxicity was repressed by SIRT1 via deacetylating forkhead box O1 (FOXO1) and the NF-κB subunit p65 on β-cells, respectively (37, 38). Multiple feedback loops are involved in SIRT signaling network. Via binding to SIRT1 promoter, p53 successfully inhibited SIRT1 transcription activity (39). FOXO3a can block the effect of p53, in consequence SIRT1 promoter will be relieved (39). Peroxisome Proliferator Activated Receptor Gamma (PPARγ) is negatively associated with SIRT1 activity (40, 41), whereas PPARα and PPARβ function in the opposite way (42, 43). MiR-199a, MiR-34a, posttranslational modification, such as phosphorylation, also affect SIRT1 activity or transcription (44–47). Taken together, SIRT1 positively regulated insulin sensitivity.

**Table 1:** Summary of sirtuin functions in diabetes development.

**Sirtuins**	**Subcellular localization**	**Enzyme actions**	**Substrates**	**Functions**
SIRT1	Nuclear Cytoplasm	Deacetylase	PTP1B, UCP2, PPARα, PPARγ, PPARγ2, p53, FOXO1, PGC-1α, NF-κB, CRTC2, SREBP	Adipogenesis↓ Lipogenesis↓ Lipolysis↑ FAO↑ Glucose uptake↑ Mitochondrial biogenesis↑ Insulin secretion↑ in the fed state Hepatic gluconeogenesis↑ in fasted state Preserve glucose homeostasis under fasted and fed conditions
SIRT2	Cytoplasm (nuclear during interphase and mitosis)	Deacetylase ADP-ribosyltransferase Demyristoylase	ERK1/2, GSK3β, p70S6 Akt, FOXO, TUG, GKRP, PEPCK1, PGC-1α	Adipogenesis↓ Lipolysis↑ FAO↑ HGU↑ Regulation of adipocyte differentiation Insulin sensitivity↑ in insulin-resistant hepatocytes, mitochondrial dysfunction↓ Insulin sensitivity↓ in skeletal muscle cells Skeletal muscle glucose uptake↓
SIRT3	Mitochondrial	Deacetylase Decrotonylase	PGC-1α, AMPK, CREB, PDH, LCAD	FAO↑ Insulin resistance↓ in skeletal muscle Regulate mitochondrial antioxidant defense enzymes, ROS↓ Urea cycle and ketogenesis in liver↑
SIRT4	Mitochondrial	ADP-ribosyltransferase Deacetylase Lipoamidase	PGC-1α, AMPK, Adenine translocator, IDE, Glutamate dehydrogenase, MCD1	Lipogenesis↑ FAO↓ Hepatic lipid accumulation↑ Insulin secretion↓ Mitochondrial biogenesis↓
SIRT5	Mitochondrial	Deacetylase Desuccinylase Deglutarylase Demalonylase	IDH2, G6P, CPS1	Ammonia detoxification↑ Regulates glucose oxidation, FAO, ROS Cellular antioxidant defense↑
SIRT6	Nuclear, associated to chromatin	Deacetylase ADP-ribosyltransferase Demyristoylase	HIF-1α, Akt, FOXO1, GCN5, IGF-1, NF-κB, GLUT1, LDH, PGK1, PFK-1	Insulin resistance↓ Gluconeogenesis↓ Mimics the effect of calorie restriction Maintains glucose homeostasis and repress mitochondrial respiration by acting as an HIF-1α corepressor
SIRT7	Nucleolar	Deacetylase	TR4/TAK1, Cd36, PPARγ,	Adipogenesis↑ Fatty acid uptake↑ Hepatic lipid accumulation↑ Triglyceride synthesis/storage↑ Controversy of fatty liver development in SIRT7 knockout mice

*Green shade infers detrimental effect, while red shade infers protective effect. The symbol ↑means increment, and ↓means decrement*.

Recently, SIRT2 has been implicated in sustaining insulin sensitivity and glucose homeostasis (48). In insulin-resistant livers and hepatocytes, SIRT2 expression was lowered, accompanied with mitochondrial dysfunction, extracellular signal-regulated kinase (ERK) activation, and amplified production of reactive oxygen species (ROS). On the contrary, insulin sensitivity and mitochondrial dysfunction was improved, and ROS generation was lessened in SIRT2-overexpressed insulin-resistant hepatocytes (49). In human peripheral blood mononuclear cells (PBMCs), insulin resistance and obesity negatively related with SIRT2 (50). As Protein Kinase B (Akt) substrates, FOXO transcription factors are deacetylated by SIRT2 (51–54). However, the function of SIRT2 in insulin signaling is still controversial. Under standard nutrient conditions, it has been suggested that Akt activation in insulin-responsive cells is mainly attributed to SIRT2, other than SIRT1 (55, 56). However, SIRT2 overexpression strengthened insulin-induced Akt/GSK3β/p70S6 signaling in HeLa cells and 3T3-L1 preadipocytes (56). It's elusive whether the phenomenon was caused by alteration of Akt acetylation status. Further investigations are needed to explore whether Akt deacetylation mediated by SIRT2 disturbs both protein binding and downstream pathway. TUG (tether, containing a UBX domain, for GLUT4) plays a role in the exocytosis of glucose transporter type 4 (GLUT4) (57), and binds with SIRT2. SIRT2 regulated the acetylation of TUG *in vitro* and *in vivo* (58). Enhanced TUG acetylation was observed in the liver of SIRT2 knockout mice, alone with greater glucose uptake and more GLUT4 storage vesicles in response to insulin (58). Altogether, SIRT2 may exert distinctive and even opposing effect in response to insulin in different tissues.

However, SIRT4 has been reported to inhibit insulin secretion (9, 59, 60). SIRT4 overexpression promotes lipogenesis and dyslipidimeia, and meanwhile diminishes FAO. All these will lead to insulin resistance (21). SIRT4 mono-ADPribosylates insulin degrading enzyme and ATP/ADP translocases in β cells, leading to downregulation of insulin secretion induced by glucose (61). SIRT4 deletion declines ATP level and low ATP level will activate 5′adenosine monophosphate-activated protein kinase (AMPK), PGC-1α and its target genes, both of which are involved in mitochondrial biogenesis and FAO. Dysregulation of AMPK signaling leads to autophagy deactivation, oxidative stress, and inflammation which are implicated in pathogenesis of insulin resistance (62).

SIRT6 plays an essential role pancreatic β-cell function and survival in mice (63). SIRT6 protected against insulin resistance and obesity induced by HFD (64). Akt phosphorylation at Ser 473 and Thr 308 were negatively regulate by SIRT6 through interfering with insulin receptors, insulin receptor substrate and various upstream molecules (65). In SIRT6 deficiency mice, increased Akt phosphorylation and activated insulin signaling is observed, yielding more glucose uptake and even hypoglycemia. Additionally, glucose induced more cell apoptosis and impaired insulin secretion severely in MIN6 β-cells in SIRT6 ablation mice. Contrariwise, SIRT6 overexpression rescued β-cell apoptosis and dysfunction (66, 67).

Hence activating SIRT1, SIRT3, and SIRT6 would be a right option to struggle with T2DM due to the repression on PTP1B and UCP2 and final increment in insulin secretion. But SIRT4 functions in a negative way in diabetes development.

## SIRTs in glucose metabolism and homeostasis

During energy restriction status, glucose will be provided by the liver to sustain normoglycemia, initially in the glycogenolysis manner and then changing to gluconeogenesis (68). In the fed condition, insulin is secreted to suppress gluconeogenic enzymes transcription including phosphoenolpyruvate carboxykinase (PEPCK1), fructose-1,6-bisphosphatase, and glucose-6-phosphatase (G6P). PGC-1α and FOXO1 can upsurge gluconeogenic enzyme genes transcription (69, 70).

SIRT1 motivates hepatic gluconeogenesis in fasting status. In contrast, SIRT1 sensitizes insulin and lowers glucose under insulin-resistant condition (71). SIRT1 also deacetylates PGC-1α, and subsequently improves gluconeogenic genes expression in the liver (71, 72), finally encouraging hepatic glucose output during fasting. SIRT2 deacetylates and stabilizes PEPCK1 under glucose deprivation conditions (73). Compromised hepatic glucose uptake (HGU) is the cause of postprandial hyperglycemia in T2DM patients (74). In diabetic mice fed with HFD, SIRT2 overexpression in liver rises HGU and alleviates glucose tolerance. In liver-specific SIRT2 knockdown mice, HGU was diminished and glucose tolerance was imbalanced. It has been reported that SIRT2 stimulates HGU probably via deacetylating K126 of glucokinase regulatory protein (GKRP) (74).

SIRT5 manipulates protein substrates which are involved in ROS management, FAO, ammonia detoxification, ketone body formation, and glucose oxidation by glutarylation, malonylation, and succinylation (75).

SIRT6 interferes with FOXO1, thus reducing gluconeogenic genes such as G6P and PEPCK (76). Hepatic gluconeogenesis was meaningfully upregulated in SIRT6 knockout mice, suggesting a compensatory reaction to hypoglycemia (77). General control non-repressed protein 5 (GCN5) acetylated PGC-1α and diminished the transcriptional activity of PGC-1α (72). SIRT6 could activate GCN5 (77). A hypoxia-inducible factor 1α (HIF-1α) inhibitor would rescue the hypoglycemia phenotype in SIRT6 deficiency mice. Mice with SIRT6 knockout in brains exhibited lower levels of insulin-like growth factor 1 (IGF-1) and growth hormone than control mice, similar to the effect achieved in whole-body SIRT6 knockout mice (78), suggesting that the central nervous system is critical in glucose metabolism.

## SIRTs in calorie restriction and exercise

Calorie restriction (CR) has been reported to postpone the onset of diabetes. During the initial phase of CR, liver gluconeogenesis is activated by pancreatic α cells-secreted glucagon, during which the cyclic AMP response-element-binding protein (CREB) and CREB-regulated transcription coactivator 2 (CRTC2) are involved. CR and exercise is beneficial for health and longevity, and genetic ablation of SIRT1, SIRT3, and SIRT6 would block the benefits provided by CR and exercise (79–83).

SIRT1 encourages FOXO1-tirggered gluconeogenesis in fasting (84). SIRT1 activators exert similar effects like CR (85, 86). However, this effect is reversed by CRTC2 deacetylation mediated by SIRT1 (84). SIRT1 then deacetylates and activates PGC-1α to facilitate gluconeogenesis (71). CRTC2 supports gluconeogenesis in the initial stage of fasting. Hepatic SIRT1 reduced CRTC2 level via deacetylation and ubiquitination, at 18 h fasting. FOXO1 accounted for gluconeogenesis after 18 h of fasting (84). Mice exposed to long-term CR (18 months) displayed SIRT2 increment in kidney and white adipose tissue (WAT) but not in brain or liver (51). Likewise, short-term fasting (24 h) also enhances protein and mRNA expression of SIRT2 in WAT (53). SIRT6 also accounted for CR function. SIRT6 knockout eliminated CR-induced life extension. SIRT6 Overexpression mimics the effects of exercise and CR in mice, and extends lifespan and health span, including reduced glucose, insulin, adipokines, cholesterol, and body weight (64, 87, 88). In addition, CR-stimulated SIRT6 repressed NF-κB pathway (89). SIRT1 also transcriptionally activated SIRT6, therefore sirtuins might work in a coordinated way to modulate each phase of calorie restriction (29).

## SIRTs in mitochondrial glycolysis and biogenesis

ATP is generated in animal cells by two principal processes, glycolysis and mitochondrial oxidative phosphorylation. T2DM, obesity, and many other aging-related disorders are characteristic of amplified oxidative damage. ROS is generally produced in mitochondria, as superoxide (O${}_{2}^{-}$) is a byproduct during electron transport system metabolism. In response to excess glucose, SIRTs will orchestrate the ratio of respiration and glycolysis, consuming energy through proton leak (90).

SIRT1 deacetylates PGC-1α, which is critical for mitochondrial function and gluconeogenesis. SIRT1 directly deacetylates and activates PGC-1α while SIRT3 enhances PGC-1α protein expression indirectly(91), through activating CREB and AMPK which accordingly increases downstream mitochondrial biogenesis targets (92–94). Significant mortality, defective thermogenesis, decreased hypoglycemia, and reduced FAO are obvious in SIRT3 knockout models (95). SIRT3 also deacetylates key genes in oxidative stress and mitochondrial antioxidant defense enzymes. The beneficial effects of SIRT3 on CR can chiefly be ascribed to inhibiting ROS (83). Actually, the alleviation in cellular oxidative stress that generates during CR is absent in SIRT3 knockout mice (82).

In contrast to SIRT3, SIRT4 inhibits mitochondrial biogenesis by suppressing PGC-1α expression. AMPK and SIRT4 interplay to retrograde PGC-1α signaling, suggesting that SIRT4 negatively manipulates mitochondrial biogenesis (96). SIRT5 has been reported to promote antioxidant defense and sustain NADPH homeostasis in cells by increasing G6P deglutarylation and isocitrate dehydrogenase 2 (IDH2) desuccinylation (97). Interestingly, SIRT6 interacts with HIF-1α to co-repress mitochondrial respiration (98). SIRT6 deficiency promoted HIF-1α activity and glycolysis by enhancing phosphofructokinase 1 (PGK1), glucose-6-phosphate isomerase, phosphoglycerate kinase (PFK-1), lactate dehydrogenase (LDH), and GLUT1 (98, 99).

## SIRTs in the metabolic homeostasis of skeletal muscle

Skeletal muscle is a critical tissue to maintain energy homeostasis. Storage of lipid metabolites and fatty acids in muscle prevents glucose uptake, finally leading to T2DM (100, 101). Induction of fatty acid β-oxidation has emerged as a hopeful method to attenuate muscle insulin resistance in muscle.

In skeletal muscle, SIRT1 expression is triggered by fasting. SIRT1 in turn deacetylates both FOXO1 and PGC-1α and facilitates fatty acid β-oxidation (91). Likewise, FAO and mitochondrial biogenesis genes are upregulated owning to SIRT1 activators SRT1720 treatment via deacetylating PGC-1α (27, 102). Moreover, SIRT1 plays a role in mitochondrial biogenesis induced by adiponectin in skeletal muscle (103).

SIRT2 negatively regulates insulin resistance and glucose uptake in C2C12 muscle cells. Akt/GSK3β signaling and glucose uptake which are driven by insulin was enlarged by inhibition of SIRT2 under insulin-resistance conditions (104). SIRT2 knockdown under insulin-resistant status enhanced insulin sensitivity in skeletal muscle cells. Nevertheless, blunt of SIRT3 and SIRT1 in C2C12 cells impairs insulin pathway and stimulates insulin resistance. Despite the fact that SIRTs possess a conserved catalytic domain, they exert a differential regulating effect on insulin resistance. SIRT3 Knockdown in muscle cells impairs insulin action and metabolic flexibility (105, 106), and muscle ability to adjust to fuel oxidation (107). SIRT3 deletion amplified acetylation of pyruvate hydrogenase (PDH), yielding declining PDH activity, and subsequent less glucose oxidation. All these gave rise to a switch to FAO, even with glucose available (106, 108).

SIRT4 negatively regulates mitochondrial biogenesis and FAO in muscles. SIRT4 regulates insulin secretion by modulating glutamate dehydrogenase. As expected, fat acid oxidative capability and mitochondrial metabolism enzymes in muscle and hepatocytes was upregulated in response to SIRT4 knockdown. In primary SIRT4 knockdown myotubes, phosphorylation of AMPK was activated, accompanied with intense cellular respiration and FAO. Moreover, protein and mRNA levels of SIRT1 were enhanced both *in vitro* and *in vivo*, largely attributed to the reduced SIRT4 levels (109).

## SIRTs in fatty acid oxidation

The development of T2DM and its complications is associated with lipid metabolism disorder. Inadequate FAO gives rise to the initiation of insulin resistance and lipid accumulation (110, 111).

SIRT1 fosters fat metabolism in liver cells, as demonstrated by the formation of fatty livers in mice with SIRT1 deletion in the liver (112, 113). SIRT1 knockout mice hardly suppressed lipogenic genes or increased FAO genes in the background of fasting (112). In normal hepatocytes, SIRT1 interacts with the PPARα response element where it deacetylates PGC-1α and increases PPARα expression, thus stimulating FAO (113, 114). Furthermore, once the fat anabolism-inducing factor PPARγ was deacetylated by SIRT1, the sterol regulatory element binding proteins (SREBPs) will be deactivated and become more susceptible to degradation (115), achieving more lipolysis. Deacetylation of PGC-1α and expression of β-oxidation genes was accordingly diminished due to SIRT2 function impairment (116).

SIRT3 plays an essential role in FAO in the mitochondria. Upon CR or fasting, SIRT3 is activated in mitochondria to stimulate FAO through inducing the deacetylation of long-chain-specific acyl coenzyme A dehydrogenase (LCAD) (95, 117). SIRT3 stimulates ketogenesis and urea cycle as well (118, 119). A chronic HFD reduced SIRT3 levels in mice, associated with LCAD function impairment and mitochondrial hyperacetylation (120).

SIRT4's effect is remarkably different from SIRT3 and SIRT1. Ablation of SIRT4 avoids steatosis during HFD (109). In addition, SIRT4 suppresses PPARα to prevent FAO, in the meantime SIRT4 inhibits malonyl CoA decarboxylase 1 (MCD1) to facilitate the synthesis of lipid (29). SIRT4 interferes with SIRT1-PPARα complex, therefore the activation effect of SIRT1 on PPARα and the inhibiting effect on FAO was abrogated.

Analogous to SIRT4, SIRT7 knockout in liver resulted in blunted triglyceride accumulation. Hepatic SIRT7 facilitated triglyceride storage/synthesis and fatty acid uptake by activating TR4/TAK1, a nuclear receptor participating in lipid metabolism. Moreover, the ubiquitin-proteasome pathway is also involved in the regulating effect of hepatic SIRT7 on lipid metabolism (121). SIRT7 also hinders TR4 degradation. TR4 involves in lipid balance by modulating monoacylglycerol O-acyltransferase 1, *Cidec*, cell death-inducing DFFA-like effector a (*Cidea*), and Cd36. SIRT7 has been reported to upsurge hepatic lipid accumulation owning to increasing Cd36 expression (121, 122). Yoshizawa et al. (121) observed that HFD failed to stimulate glucose intolerance, obesity, or fatty liver in SIRT7 knockout mice. The conclusion is controversial to the result that SIRT7 knockout promoted fatty liver development (123, 124). Compared with SIRT1, SIRT7 displays distinctive effect on maintaining liver lipid homeostasisa. Lipid storage is raised by SIRT7 by suppressing PPARα, like SIRT4 (125).

## SIRTs in the metabolic homeostasis of adipocyte

Regarded as a storage compartment for fatty lipids, adipose tissue also serves as an important modulator for energy homeostasis, vascular remodeling endocrine functions, insulin sensitivity, and innate immune response (126). Adipokines, such as leptin and adiponectin, are systemically regulated by white adipose tissue (WAT). There is convincing evidence supporting that adiponectin derived from adipocyte plays an essential part in insulin resistance (127, 128). Moreover, adiponectin sustains glucose homeostasis and protects against diabetes and obesity.

Numerous SIRT1 benefits take place in WAT (129). SIRT1 inhibits lipogenesis and stimulates fat mobilization in adipocytes from WAT, via suppressing PPARγ. Eventually, peripheral utilization of the fat storages is raised (113). White fat can be switched to metabolically active brown fat due to SIRT1 deacetylation on PPARγ (130). Conversely, SIRT1 can be cleaved in WAT by caspase I and inflammasome which is activated by HFD (19).

SIRT1 knockdown reduced WAT mass in rats. The mRNA contents of PPARγ2 and PPARγ, both of which were adipogenic genes, are abridged in adipose, driving adipocyte differentiation and adipose loss. Adipocytes-specific SIRT1 knockdown led to lower plasma concentrations of adiponectin and leptin. In adipose of obese individuals, mRNA levels of SIRT1 were lower in than those of control group. In the opposite, obese patients have higher SIRT7 expressions in adipose. SIRT7 and SIRT1 promoters' methylation status are not closely associated with the upregulation or downregulation of their mRNA levels induced by obesity. In visceral adipose tissue (VAT) of obese patients, the content of miR-181a-3p and miR-34a-5p negatively associated with SIRT1 levels. In contrary, the expression of miR-125b-5p and miR-125a-5p negatively correlated with SIRT7 in VAT of slim subjects (131). Furthermore, MiR-93 impedes the metabolic target SIRT7 (132).

SIRT7 has been identified as a chief driver of adipogenesis by inducing differentiation and maturation of early adipocyte precursors. PPARγ is adipogenic and its expression is reduced in the WAT of mice with SIRT7 deletion (132), designating that SIRT7 stimulates adipogenesis. SIRT7 can remove long-chain fatty acyl groups more efficiently than removing acetyl groups (133, 134).

Inhibited SIRT2 expression and amplified HIF-1α expression are observed in VAT from obese individuals. HIF-1α hinders adipocyte-mediated fatty acid catabolism by blocking SIRT2-PGC-1α pathway, thereby favoring the progression of obesity (116). Diet-induced obesity was strengthened in SIRT6 knockout mice, principally attributed to hypertrophy of adipocyte other than differentiation of abnormal adipocyte (135).

## SIRTs in neurons under diabetic conditions

In the anorexigenic proopiomelanocortin (POMC) neurons, SIRT1 is critical in preserving normal energy expenditure. POMC neurons-specific SIRT1 knockout mice are vulnerable to diet-induced obesity (136). SIRT1 is also defensive against diabetes and obesity in the steroidogenic factor 1 neurons (137). Additionally, the peptide release of orexigenic agouti is suppressed by SIRT1 via interacting with FOXO1 (138, 139). Nevertheless, SIRT1 ablation in neurons was related with insulin secretion in hypothalamic neurons (140).

SIRT2, PGC-1α, and P-AMPK declined dramatically in diabetic cortex. AMPK/SIRT/PGC-1α pathway, which mediates antioxidant abilities and mitochondrial biogenesis, is involved in cortex neurodegeneration progression under diabetic conditions (141). SIRT6 and SIRT2 expression were meaningfully reduced in the neural stem or embryo cells from pre-gestational maternal diabetes. Superoxide dismutase 1 (SOD1) mimetic and overexpression rescued the decrement of SIRT6 and SIRT2 in the diabetic embryopathy mouse model. Histone acetylation caused by Sirtuin decrement might participates in neural tube defects induced by diabetes.

## SIRTs polymorphisms in diabetes

While abundant data point to the essential role of SIRTs activities, there are genetic polymorphisms of SIRT1 and SIRT2 concerning diabetes. rs10509291 and rs7896005 in SIRT1 genes are associated with T2DM development as well as reduced acute insulin response (142). In a report about Japanese patients with T2DM, four single nucleotide polymorphisms (SNPs) in SIRT1 that were positively correlated with diabetic nephropathy, and a haplotype containing the SNPs within SIRT1 locus had a stronger association (143). Moreover, SIRT1 mutation has been reported to link with autoimmune diabetes. Type 1 diabetes mellitus (T1DM) is an autoimmune disease characteristic of autoimmune-mediated β cell destruction. Lately, exome, and direct sequencing recognized a T-to-C exchange in exon 1 of *SIRT1* in a patient diagnosed with T1DM, corresponding to a leucine-to-proline mutation at residue 107. It is speculated that the SIRT1 L107P mutation, located within the N-terminal protein-binding domain, could also disturb the oligomerization and activity (20). Furthermore, the prenatally famine-exposed kids, who have minor alleles of SIRT1 gene (GA and AA of rs1467568 and AG and GG of rs7895833), have a lower risk for T2DM in adult life (144). DNA sequence variants (DSVs), including g.38900237G > A, g.38900359C > T, g.38900561C > T, and g.38900912G > T, might upsurge SIRT2 gene promoter activity and SIRT2 levels, contributing to T2DM as a risk factor (50).

## Clinical trials of SIRTs activators in diabetes: current evidence

As mentioned above, SIRTs activators exert positive effects in neurodegenerative, and metabolism diseases. Identification of SIRT1 activators for T2DM treatment has become immediate areas of research focus, such as metformin, resveratrol, resveratrol derivatives (Resveratrol aliphatic acids, acetylated derivatives, 3,3′,4,4′,5,5′-hexahydroxystilbene) and polyphenols (quercetin, piceatannol, fisetin, pinosylvin, and butein) (145). Metformin is the recommended first-line oral glucose-lowering drug initiated to control hyperglycemia in T2DM, a synthetic dimethyl biguanid (146). SIRT1 level rises after metformin treatment. SIRT1 is obviously entangled into the mechanism of metformin action (147). In the largest Randomized Controlled Trial inspecting metformin for diabetes prevention (148, 149), 1073 subjects from 27 USA medical centers took metformin (850 mg twice every day) or placebo (*n* = 1082). As compared with placebo, diabetes occurrence was considerably decreased by 31% (95% *CI* = 17%, 43%) in the metformin group after almost 3 years follow-up.

When it comes to another potent SIRT activator resveratrol, SIRTris Pharmaceuticals had launched an oral resveratrol formulation (SRT501), which has entered Phase III clinical trials for T2DM therapy (150). Effects of trans-resveratrol extract from Polygonum in patients with type 2 diabetes has completed Phase I clinical trials, with trial number NCT01677611 (151). In a dose-escalation Phase I trial, resveratrol clearance (5 g in a single dose) was rapid, and urine excretion reached 77% within 4 h, signifying that derivatives structure optimization with longer half-life is in great need (152). Moreover, derivatives SRT-2183, SRT-1720, and SRT-1460 are also discovered. But SRT-1720 was terminated owing to limited effect. SRT-2104 was more potent and the Phase II clinical trials has completed successfully (153). The pharmacokinetics and safety study of SRT2379 evaluated in healthy male volunteers has finished the Phase I clinical trials (154).

## Conclusion

SIRTs play a noticeable role in modulating insulin resistance and glucose uptake in adipose tissue, liver, and muscle. SIRT1, SIRT2, SIRT3, and SIRT6 has been implicated to positively sustain insulin sensitivity and glucose homeostasis, rendering them attractive potential drug targets. While SIRT4 and SIRT7 negatively regulate insulin secretion and FAO.

Specifically, SIRT1 enhances fat catabolism in adipose tissue, skeletal muscle and liver by modifying the activity of PPARα, PPARγ, and PGC-1α. Apart from inducing fat catabolism, SIRT1 also promotes FAO, mitochondrial oxidative capacity and energy expenditure in fat tissue and skeletal muscle, not only through direct activation of PPARα, but also through secondary activation of AMPK and PPARα by SIRT1-mediated adiponectin synthesis. SIRT1 prevents lipogenesis and motivates free fatty acid release by inhibiting SREBP and PPARγ. SIRT1 exhibits conflicting effects on maintaining glucose homeostasis under fed and fasted conditions. In fed condition, SIRT1 reinforces pancreatic insulin secretion. In fasted status, SIRT1 promotes hepatic gluconeogenesis by deacetylating FOXO1 and PGC-1α. SIRT1 exerts insulin-sensitizing effect by inhibiting PTP1B and UCP2 expression and regulating adiponectin synthesis. SIRT2 increases insulin sensitivity in insulin-resistant hepatocytes, while decreases insulin sensitivity in skeletal muscle cells. Nevertheless, there is very limited literature on SIRT5 enzyme activity until the recent finding as it can remove succinyl or malonyl groups, and this action resembles deacetylation. SIRT5 is broadly expressed, but SIRT5-deficient mice are healthy, fertile, and without major clinical phenotype (155), inferring that SIRT5 is not indispensable for metabolic homeostasis at least under basal conditions. SIRT6 supports pancreatic β-cell function and sustains glucose homeostasis by acting as a HIF-1α corepressor. Conversely, SIRT4 and SIRT7 exhibited negative effect on diabetes therapy, such as aggravating lipogenesis, and inhibiting insulin secretion.

The majority of sirtuins isoforms are protective on diabetes and a minority appears to be detrimental, but the antagonism effect on the whole body remains elusive. Although several metabolic pathways and targets have been proposed to mediate SIRTs function on T2DM, some outstanding questions need to be resolved. Moreover, do these SIRTs act independently or synergistically on diabetes? How do they communicate for cooperative actions in cells? SIRTs are regulated by protein-protein interactions and microRNAs at the level of translation and transcription (156), however, little is known about the epigenetic mechanisms modifying sirtuins. In some cases, sirtuins isoforms regulate certain essential enzymes in an opposite direction. For instance, PDH can be activated through deacetylation by SIRT3 (106), while both delipoamidating by SIRT4 or desuccinylating by SIRT5 inhibited PDH activity (157, 158), and the question that which effect will win out is quite a puzzle. Furthermore, when SIRTs display both ADP-ribosyltransferase and deacetylase activity, the circumstances that decide the predominant activity need to be determined.

A plenty of clinical trials has been carried out, including resveratrol, metformin, and other SIRT activators. It is possible that in the foreseeable future one or more SIRT activators will be approved for diabetes therapy. As a well-known pharmaceutical preparation, the widespread usage of metformin facilitates the recruit of a large randomized controlled trial. Metformin has been regarded as the most promising candidate. But it's a little harder to explore the natural compounds in a large scale, such as curcumin, berberine, and genistein. It is attributed to the complexity in reducing batches variability of supplements and the difficulty in evaluating dietary intake in observational studies (159–161). Long-term, outcomes-based placebo-controlled rigorous clinical trials would be crucial to confirm the function of SIRT activators on diabetes.

## Author contributions

JS and LF drafted the manuscript. All authors contributed in the discussion section, and approved it for publication.

### Conflict of interest statement

The authors declare that the research was conducted in the absence of any commercial or financial relationships that could be construed as a potential conflict of interest.

## References

[1] Imai S Armstrong CM Kaeberlein M Guarente L. Transcriptional silencing and longevity protein Sir2 is an NAD-dependent histone deacetylase. Nature (2000) 403:795–800. 10.1038/35001622 10693811

[2] Chang HC Guarente L. SIRT1 and other sirtuins in metabolism. Trends Endocrinol Metab. (2014) 25:138–45. 10.1016/j.tem.2013.12.001 24388149 PMC3943707

[3] Haigis MC Sinclair DA. Mammalian sirtuins: biological insights and disease relevance. Annu Rev Pathol. (2010) 5:253–95. 10.1146/annurev.pathol.4.110807.092250 20078221 PMC2866163

[4] Parenti MD Bruzzone S Nencioni A Del Rio A. Selectivity hot-spots of sirtuin catalytic cores. Mol Biosyst. (2015) 11:2263–72. 10.1039/c5mb00205b 26061123

[5] Schuetz A Min J Antoshenko T Wang CL Allali-Hassani A Dong A. Structural basis of inhibition of the human NAD+-dependent deacetylase SIRT5 by suramin. Structure (2007) 15:377–89. 10.1016/j.str.2007.02.002 17355872

[6] Houtkooper RH Pirinen E Auwerx J. Sirtuins as regulators of metabolism and healthspan. Nat Rev Mol Cell Biol. (2012) 13:225–238. 10.1038/nrm3293 22395773 PMC4872805

[7] Rauh D Fischer F Gertz M Lakshminarasimhan M Bergbrede T Aladini F. An acetylome peptide microarray reveals specificities and deacetylation substrates for all human sirtuin isoforms. Nat Commun. (2013) 4:2327. 10.1038/ncomms3327 23995836

[8] Teng YB Jing H Aramsangtienchai P He B Khan S Hu J. Efficient demyristoylase activity of SIRT2 revealed by kinetic and structural studies. Sci Rep. (2015) 5:8529. 10.1038/srep08529 25704306 PMC4894398

[9] Haigis MC Mostoslavsky R Haigis KM Fahie K Christodoulou DC Murphy AJ. SIRT4 inhibits glutamate dehydrogenase and opposes the effects of calorie restriction in pancreatic beta cells. Cell (2006) 126:941–54. 10.1016/j.cell.2006.06.057 16959573

[10] Osborne B Bentley NL Montgomery MK Turner N. The role of mitochondrial sirtuins in health and disease. Free Radic Biol Med. (2016) 100:164–174. 10.1016/j.freeradbiomed.2016.04.197 27164052

[11] Du J Zhou Y Su X Yu JJ Khan S Jiang H. Sirt5 is a NAD-dependent protein lysine demalonylase and desuccinylase. Science (2011) 334:806–9. 10.1126/science.1207861 22076378 PMC3217313

[12] Nakagawa T Lomb DJ Haigis MC Guarente L. SIRT5 deacetylates carbamoyl phosphate synthetase 1 and regulates the urea cycle. Cell (2009) 137:560–70. 10.1016/j.cell.2009.02.026 19410549 PMC2698666

[13] Kuang J Chen L Tang Q Zhang J Li Y He J. The role of Sirt6 in obesity and diabetes. Front Physiol. (2018) 9:135. 10.3389/fphys.2018.00135 29535637 PMC5835030

[14] Ford E Voit R Liszt G Magin C Grummt I Guarente L. Mammalian Sir2 homolog SIRT7 is an activator of RNA polymerase I transcription. Genes Dev. (2006) 20:1075–80. 10.1101/gad.1399706 16618798 PMC1472467

[15] Tsai YC Greco TM Boonmee A Miteva Y Cristea IM Functional proteomics establishes the interaction of SIRT7 with chromatin remodeling complexes and expands its role in regulation of RNA polymerase I transcription. Mol Cell Proteomics (2012) 11:60–76. 10.1074/mcp.A111.015156 22586326 PMC3418843

[16] Zhou Z Sun B Li X Zhu C. DNA methylation landscapes in the pathogenesis of type 2 diabetes mellitus. Nutr Metab. (2018) 15:47. 10.1186/s12986-018-0283-x 29988495 PMC6025823

[17] Freeman AM Pennings N Insulin resistance. In: Treasure Island. Lumberton, NC: StatPearls (2018), p. 34.

[18] Kahn SE Cooper ME Del Prato S. Pathophysiology and treatment of type 2 diabetes: perspectives on the past, present, and future. Lancet (2014) 383:1068–83. 10.1016/S0140-6736(13)62154-6 24315620 PMC4226760

[19] Chalkiadaki A Guarente L. High-fat diet triggers inflammation-induced cleavage of SIRT1 in adipose tissue to promote metabolic dysfunction. Cell Metab. (2012) 16:180–8. 10.1016/j.cmet.2012.07.003 22883230 PMC3539750

[20] Biason-Lauber A Boni-Schnetzler M Hubbard BP Bouzakri K Brunner A Cavelti-Weder C. Identification of a SIRT1 mutation in a family with type 1 diabetes. Cell Metab. (2013) 17:448–455. 10.1016/j.cmet.2013.02.001 23473037 PMC3746172

[21] Choubey SK Prabhu D Nachiappan M Biswal J Jeyakanthan J. Molecular modeling, dynamics studies and density functional theory approaches to identify potential inhibitors of SIRT4 protein from Homo sapiens: a novel target for the treatment of type 2 diabetes. J Biomol Struct Dyn. (2017) 35:3316–3329. 10.1080/07391102.2016.1254117 27800715

[22] Song R Xu W Chen Y Li Z Zeng Y Fu Y. The expression of Sirtuins 1 and 4 in peripheral blood leukocytes from patients with type 2 diabetes. Eur J Histochem. (2011) 55:e10. 10.4081/ejh.2011.e10 21556116 PMC3167349

[23] Arab Sadeghabadi Z Ziamajidi N Abbasalipourkabir R Mohseni R Garlic (*Allium sativum*) increases SIRT1 and SIRT2 gene expressions in the kidney and liver tissues of STZ– and STZ+niacinamide-induced diabetic rats. J Basic Clin Physiol Pharmacol. (2018) 29:463–7. 10.1515/jbcpp-2017-0079 29672269

[24] Pedersen SB Olholm J Paulsen SK Bennetzen MF Richelsen B. Low Sirt1 expression, which is upregulated by fasting, in human adipose tissue from obese women. Int J Obes. (2008) 32:1250–5. 10.1038/ijo.2008.78 18560370

[25] Costa Cdos S Hammes TO Rohden F Margis R Bortolotto JW Padoin AV SIRT1 transcription is decreased in visceral adipose tissue of morbidly obese patients with severe hepatic steatosis. Obes Surg. (2010) 20:633–9. 10.1007/s11695-009-0052-z 20033348

[26] Baur JA Pearson KJ Price NL Jamieson HA Lerin C Kalra A. Resveratrol improves health and survival of mice on a high-calorie diet. Nature (2006) 444:337–42. 10.1038/nature05354 17086191 PMC4990206

[27] Lagouge M Argmann C Gerhart-Hines Z Meziane H Lerin C Daussin F. Resveratrol improves mitochondrial function and protects against metabolic disease by activating SIRT1 and PGC-1alpha. Cell (2006) 127:1109–22. 10.1016/j.cell.2006.11.013 17112576

[28] Bordone L Cohen D Robinson A Motta MC van Veen E Czopik A. SIRT1 transgenic mice show phenotypes resembling calorie restriction. Aging Cell (2007) 6:759–67. 10.1111/j.1474-9726.2007.00335.x 17877786

[29] Kim HS Xiao C Wang RH Lahusen T Xu X Vassilopoulos A. Hepatic-specific disruption of SIRT6 in mice results in fatty liver formation due to enhanced glycolysis and triglyceride synthesis. Cell Metab. (2010) 12:224–36. 10.1016/j.cmet.2010.06.009 20816089 PMC2935915

[30] Saltiel AR Kahn CR. Insulin signalling and the regulation of glucose and lipid metabolism. Nature (2001) 414:799–806. 10.1038/414799a 11742412

[31] Jukarainen S Heinonen S Ramo JT Rinnankoski-Tuikka R Rappou E Tummers M. Obesity is associated with low NAD(+)/SIRT pathway expression in adipose tissue of BMI-discordant monozygotic twins. J Clin Endocrinol Metab. (2016) 101:275–83. 10.1210/jc.2015-3095 26574954

[32] Sun C Zhang F Ge X Yan T Chen X Shi X. SIRT1 improves insulin sensitivity under insulin-resistant conditions by repressing PTP1B. Cell Metab. (2007) 6:307–19. 10.1016/j.cmet.2007.08.014 17908559

[33] Elchebly M Payette P Michaliszyn E Cromlish W Collins S Loy AL. Increased insulin sensitivity and obesity resistance in mice lacking the protein tyrosine phosphatase-1B gene. Science (1999) 283:1544–8. 10066179 10.1126/science.283.5407.1544

[34] Moynihan KA Grimm AA Plueger MM Bernal-Mizrachi E Ford E Cras-Meneur C. Increased dosage of mammalian Sir2 in pancreatic beta cells enhances glucose-stimulated insulin secretion in mice. Cell Metab. (2005) 2:105–17. 10.1016/j.cmet.2005.07.001 16098828

[35] Zhang CY Baffy G Perret P Krauss S Peroni O Grujic D. Uncoupling protein-2 negatively regulates insulin secretion and is a major link between obesity, beta cell dysfunction, and type 2 diabetes. Cell (2001) 105:745–55. 10.1016/S0092-8674(01)00378-6 11440717

[36] Bordone L Motta MC Picard F Robinson A Jhala US Apfeld J. Sirt1 regulates insulin secretion by repressing UCP2 in pancreatic beta cells. PLoS Biol. (2006) 4:e31. 10.1371/journal.pbio.0040031 16366736 PMC1318478

[37] Kitamura YI Kitamura T Kruse JP Raum JC Stein R Gu W. FoxO1 protects against pancreatic beta cell failure through NeuroD and MafA induction. Cell Metab. (2005) 2:153–63. 10.1016/j.cmet.2005.08.004 16154098

[38] Lee JH Song MY Song EK Kim EK Moon WS Han MK. Overexpression of SIRT1 protects pancreatic beta-cells against cytokine toxicity by suppressing the nuclear factor-kappaB signaling pathway. Diabetes (2009) 58:344–51. 10.2337/db07-1795 19008341 PMC2628607

[39] Nemoto S Fergusson MM Finkel T. Nutrient availability regulates SIRT1 through a forkhead-dependent pathway. Science (2004) 306:2105–8. 10.1126/science.1101731 15604409

[40] Bai P Canto C Brunyanszki A Huber A Szanto M Cen Y. PARP-2 regulates SIRT1 expression and whole-body energy expenditure. Cell Metab. (2011) 13:450–460. 10.1016/j.cmet.2011.03.013 21459329 PMC3108571

[41] Han L Zhou R Niu J McNutt MA Wang P Tong T. SIRT1 is regulated by a PPAR{gamma}-SIRT1 negative feedback loop associated with senescence. Nucleic Acids Res. (2010) 38:7458–71. 10.1093/nar/gkq609 20660480 PMC2995042

[42] Hayashida S Arimoto A Kuramoto Y Kozako T Honda S Shimeno H. Fasting promotes the expression of SIRT1, an NAD+ -dependent protein deacetylase, via activation of PPARalpha in mice. Mol Cell Biochem. (2010) 339:285–92. 10.1007/s11010-010-0391-z 20148352

[43] Okazaki M Iwasaki Y Nishiyama M Taguchi T Tsugita M Nakayama S. PPARβ/δ regulates the human SIRT1 gene transcription via Sp1. Endocr J. (2010) 57:403–13. 10.1507/endocrj.K10E-004 20160399

[44] Yamakuchi M Ferlito M Lowenstein CJ. miR-34a repression of SIRT1 regulates apoptosis. Proc Natl Acad Sci USA. (2008) 105:13421–6. 10.1073/pnas.0801613105 18755897 PMC2533205

[45] Rane S He M Sayed D Vashistha H Malhotra A Sadoshima J. Downregulation of miR-199a derepresses hypoxia-inducible factor-1alpha and Sirtuin 1 and recapitulates hypoxia preconditioning in cardiac myocytes. Circ Res. (2009) 104:879–86. 10.1161/CIRCRESAHA.108.193102 19265035 PMC3332328

[46] Sasaki T Maier B Koclega KD Chruszcz M Gluba W Stukenberg PT. Phosphorylation regulates SIRT1 function. PLoS ONE (2008) 3:e4020. 10.1371/journal.pone.0004020 19107194 PMC2602979

[47] Conrad E Polonio-Vallon T Meister M Matt S Bitomsky N Herbel C. HIPK2 restricts SIRT1 activity upon severe DNA damage by a phosphorylation-controlled mechanism. Cell Death Differ. (2016) 23:110–22. 10.1038/cdd.2015.75 26113041 PMC4815982

[48] Gomes P Fleming Outeiro T Cavadas C. Emerging role of sirtuin 2 in the regulation of mammalian metabolism. Trends Pharmacol Sci. (2015) 36:756–68. 10.1016/j.tips.2015.08.001 26538315

[49] Lemos V de Oliveira RM Naia L Szego E Ramos E Pinho S. The NAD+-dependent deacetylase SIRT2 attenuates oxidative stress and mitochondrial dysfunction and improves insulin sensitivity in hepatocytes. Hum Mol Genet. (2017) 26:4105–17. 10.1093/hmg/ddx298 28973648

[50] Liu T Yang W Pang S Yu S Yan B. Functional genetic variants within the SIRT2 gene promoter in type 2 diabetes mellitus. Diabetes Res Clin Pract. (2018) 137:200–07. 10.1016/j.diabres.2018.01.012 29371109

[51] Wang F Nguyen M Qin FX Tong Q. SIRT2 deacetylates FOXO3a in response to oxidative stress and caloric restriction. Aging Cell (2007) 6:505–14. 10.1111/j.1474-9726.2007.00304.x 17521387

[52] Jing E Gesta S Kahn CR. SIRT2 regulates adipocyte differentiation through FoxO1 acetylation/deacetylation. Cell Metab. (2007) 6:105–14. 10.1016/j.cmet.2007.07.003 17681146 PMC2083635

[53] Wang F Tong Q. SIRT2 suppresses adipocyte differentiation by deacetylating FOXO1 and enhancing FOXO1's repressive interaction with PPARgamma. Mol Biol Cell (2009) 20:801–8. 10.1091/mbc.E08-06-0647 19037106 PMC2633403

[54] Motta MC Divecha N Lemieux M Kamel C Chen D Gu W. Mammalian SIRT1 represses forkhead transcription factors. Cell (2004) 116:551–63. 10.1016/S0092-8674(04)00126-6 14980222

[55] Chen J Chan AW To KF Chen W Zhang Z Ren J. SIRT2 overexpression in hepatocellular carcinoma mediates epithelial to mesenchymal transition by protein kinase B/glycogen synthase kinase-3beta/beta-catenin signaling. Hepatology (2013) 57:2287–98. 10.1002/hep.26278 23348706

[56] Ramakrishnan G Davaakhuu G Kaplun L Chung WC Rana A Atfi A. Sirt2 deacetylase is a novel AKT binding partner critical for AKT activation by insulin. J Biol Chem. (2014) 289:6054–66. 10.1074/jbc.M113.537266 24446434 PMC3937672

[57] Bogan JS Hendon N McKee AE Tsao TS Lodish HF. Functional cloning of TUG as a regulator of GLUT4 glucose transporter trafficking. Nature (2003) 425:727–33. 10.1038/nature01989 14562105

[58] Belman JP Bian RR Habtemichael EN Li DT Jurczak MJ Alcazar-Roman A. Acetylation of TUG protein promotes the accumulation of GLUT4 glucose transporters in an insulin-responsive intracellular compartment. J Biol Chem. (2015) 290:4447–63. 10.1074/jbc.M114.603977 25561724 PMC4326849

[59] Hutton JC Sener A Malaisse WJ. Interaction of branched chain amino acids and keto acids upon pancreatic islet metabolism and insulin secretion. J Biol Chem. (1980) 255:7340–6. 6993486

[60] Huynh FK Hu X Lin Z Johnson JD Hirschey MD. Loss of sirtuin 4 leads to elevated glucose- and leucine-stimulated insulin levels and accelerated age-induced insulin resistance in multiple murine genetic backgrounds. J Inherit Metab Dis. (2018) 41:59–72. 10.1007/s10545-017-0069-8 28726069 PMC5775063

[61] Ahuja N Schwer B Carobbio S Waltregny D North BJ Castronovo V. Regulation of insulin secretion by SIRT4, a mitochondrial ADP-ribosyltransferase. J Biol Chem. (2007) 282:33583–92. 10.1074/jbc.M705488200 17715127

[62] Li SZheng W. Mammalian sirtuins SIRT4 and SIRT7. Prog Mol Biol Transl Sci. (2018) 154:147–68. 10.1016/bs.pmbts.2017.11.001 29413176

[63] Qin K Zhang N Zhang Z Nipper M Zhu Z Leighton J. SIRT6-mediated transcriptional suppression of Txnip is critical for pancreatic beta cell function and survival in mice. Diabetologia (2018) 61:906–18. 10.1007/s00125-017-4542-6 29322219 PMC6203439

[64] Kanfi Y Peshti V Gil R Naiman S Nahum L Levin E. SIRT6 protects against pathological damage caused by diet-induced obesity. Aging Cell (2010) 9:162–73. 10.1111/j.1474-9726.2009.00544.x 20047575

[65] Xiao C Kim HS Lahusen T Wang RH Xu X Gavrilova O. SIRT6 deficiency results in severe hypoglycemia by enhancing both basal and insulin-stimulated glucose uptake in mice. J Biol Chem. (2010) 285:36776–84. 10.1074/jbc.M110.168039 20847051 PMC2978606

[66] Song MY Wang J Ka SO Bae EJ Park BH. Insulin secretion impairment in Sirt6 knockout pancreatic beta cells is mediated by suppression of the FoxO1-Pdx1-Glut2 pathway. Sci Rep. (2016) 6:30321. 10.1038/srep30321 27457971 PMC4960548

[67] Xiong X Sun X Wang Q Qian X Zhang Y Pan X. SIRT6 protects against palmitate-induced pancreatic beta-cell dysfunction and apoptosis. J Endocrinol. (2016) 231:159–65. 10.1530/JOE-16-0317 27601447 PMC5365398

[68] Bouche C Serdy S Kahn CR Goldfine AB. The cellular fate of glucose and its relevance in type 2 diabetes. Endocr Rev. (2004) 25:807–30. 10.1210/er.2003-0026 15466941

[69] Koo SH Flechner L Qi L Zhang X Screaton RA Jeffries S. The CREB coactivator TORC2 is a key regulator of fasting glucose metabolism. Nature (2005) 437:1109–11. 10.1038/nature03967 16148943

[70] Puigserver P Rhee J Donovan J Walkey CJ Yoon JC Oriente F. Insulin-regulated hepatic gluconeogenesis through FOXO1-PGC-1alpha interaction. Nature (2003) 423:550–5. 10.1038/nature01667 12754525

[71] Rodgers JT Lerin C Haas W Gygi SP Spiegelman BM Puigserver P. Nutrient control of glucose homeostasis through a complex of PGC-1alpha and SIRT1. Nature (2005) 434:113–8. 10.1038/nature03354 15744310

[72] Lerin C Rodgers JT Kalume DE Kim SH Pandey A Puigserver P. GCN5 acetyltransferase complex controls glucose metabolism through transcriptional repression of PGC-1alpha. Cell Metab. (2006) 3:429–38. 10.1016/j.cmet.2006.04.013 16753578

[73] Jiang W Wang S Xiao M Lin Y Zhou L Lei Q. Acetylation regulates gluconeogenesis by promoting PEPCK1 degradation via recruiting the UBR5 ubiquitin ligase. Mol Cell (2011) 43:33–44. 10.1016/j.molcel.2011.04.028 21726808 PMC3962309

[74] Watanabe H Inaba Y Kimura K Matsumoto M Kaneko S Kasuga M. Sirt2 facilitates hepatic glucose uptake by deacetylating glucokinase regulatory protein. Nat Commun. (2018) 9:30. 10.1038/s41467-017-02537-6 29296001 PMC5750207

[75] Kumar S Lombard DB. Generation and purification of catalytically active recombinant sirtuin5 (SIRT5) protein. Methods Mol Biol. (2016) 1436:241–57. 10.1007/978-1-4939-3667-0_16 27246219 PMC4890613

[76] Zhang P Tu B Wang H Cao Z Tang M Zhang C. Tumor suppressor p53 cooperates with SIRT6 to regulate gluconeogenesis by promoting FoxO1 nuclear exclusion. Proc Natl Acad Sci USA. (2014) 111:10684–9. 10.1073/pnas.1411026111 25009184 PMC4115576

[77] Dominy JE Jr Lee Y Jedrychowski MP Chim H Jurczak MJ Camporez JP. The deacetylase Sirt6 activates the acetyltransferase GCN5 and suppresses hepatic gluconeogenesis. Mol Cell (2012) 48:900–13. 10.1016/j.molcel.2012.09.030 23142079 PMC3534905

[78] Schwer B Schumacher B Lombard DB Xiao C Kurtev MV Gao J. Neural sirtuin 6 (Sirt6) ablation attenuates somatic growth and causes obesity. Proc Natl Acad Sci USA. (2010) 107:21790–4. 10.1073/pnas.1016306107 21098266 PMC3003110

[79] Boily G Seifert EL Bevilacqua L He XH Sabourin G Estey C. SirT1 regulates energy metabolism and response to caloric restriction in mice. PLoS ONE (2008) 3:e1759. 10.1371/journal.pone.0001759 18335035 PMC2258149

[80] Chen D Steele AD Lindquist S Guarente L. Increase in activity during calorie restriction requires Sirt1. Science (2005) 310:1641. 10.1126/science.1118357 16339438

[81] Hebert AS Dittenhafer-Reed KE Yu W Bailey DJ Selen ES Boersma MD. Calorie restriction and SIRT3 trigger global reprogramming of the mitochondrial protein acetylome. Mol Cell (2013) 49:186–99. 10.1016/j.molcel.2012.10.024 23201123 PMC3704155

[82] Qiu X Brown K Hirschey MD Verdin E Chen D. Calorie restriction reduces oxidative stress by SIRT3-mediated SOD2 activation. Cell Metab. (2010) 12:662–7. 10.1016/j.cmet.2010.11.015 21109198

[83] Someya S Yu W Hallows WC Xu J Vann JM Leeuwenburgh C. Sirt3 mediates reduction of oxidative damage and prevention of age-related hearing loss under caloric restriction. Cell (2010) 143:802–12. 10.1016/j.cell.2010.10.002 21094524 PMC3018849

[84] Liu Y Dentin R Chen D Hedrick S Ravnskjaer K Schenk S. A fasting inducible switch modulates gluconeogenesis via activator/coactivator exchange. Nature (2008) 456:269–73. 10.1038/nature07349 18849969 PMC2597669

[85] Milne JC Lambert PD Schenk S Carney DP Smith JJ Gagne DJ. Small molecule activators of SIRT1 as therapeutics for the treatment of type 2 diabetes. Nature (2007) 450:712–6. 10.1038/nature06261 18046409 PMC2753457

[86] Barger JL Kayo T Vann JM Arias EB Wang J Hacker TA. A low dose of dietary resveratrol partially mimics caloric restriction and retards aging parameters in mice. PLoS ONE (2008) 3:e2264. 10.1371/journal.pone.0002264 18523577 PMC2386967

[87] Kanfi Y Naiman S Amir G Peshti V Zinman G Nahum L. The sirtuin SIRT6 regulates lifespan in male mice. Nature (2012) 483:218–21. 10.1038/nature10815 22367546

[88] Satoh A Brace CS Ben-Josef G West T Wozniak DF Holtzman DM. SIRT1 promotes the central adaptive response to diet restriction through activation of the dorsomedial and lateral nuclei of the hypothalamus. J Neurosci. (2010) 30:10220–32. 10.1523/JNEUROSCI.1385-10.2010 20668205 PMC2922851

[89] Zhang HJ Zhang XF Ma ZM Pan LL Chen Z Han HW. Irisin is inversely associated with intrahepatic triglyceride contents in obese adults. J Hepatol. (2013) 59:557–62. 10.1016/j.jhep.2013.04.030 23665283

[90] de Moura MB Uppala R Zhang Y Van Houten B Goetzman ES Overexpression of mitochondrial sirtuins alters glycolysis and mitochondrial function in HEK293 cells. PLoS ONE (2014) 9:e106028 10.1371/journal.pone.0106028 25165814 PMC4148395

[91] Gerhart-Hines Z Rodgers JT Bare O Lerin C Kim SH Mostoslavsky R. Metabolic control of muscle mitochondrial function and fatty acid oxidation through SIRT1/PGC-1alpha. EMBO J. (2007) 26:1913–23. 10.1038/sj.emboj.7601633 17347648 PMC1847661

[92] Giralt A Hondares E Villena JA Ribas F Diaz-Delfin J Giralt M. Peroxisome proliferator-activated receptor-gamma coactivator-1alpha controls transcription of the Sirt3 gene, an essential component of the thermogenic brown adipocyte phenotype. J Biol Chem. (2011) 286:16958–66. 10.1074/jbc.M110.202390 21454513 PMC3089539

[93] Kong X Wang R Xue Y Liu X Zhang H Chen Y. Sirtuin 3, a new target of PGC-1alpha, plays an important role in the suppression of ROS and mitochondrial biogenesis. PLoS ONE (2010) 5:e11707. 10.1371/journal.pone.0011707 20661474 PMC2908542

[94] Shi T Wang F Stieren E Tong Q. SIRT3, a mitochondrial sirtuin deacetylase, regulates mitochondrial function and thermogenesis in brown adipocytes. J Biol Chem. (2005) 280:13560–7. 10.1074/jbc.M414670200 15653680

[95] Hirschey MD Shimazu T Goetzman E Jing E Schwer B Lombard DB. SIRT3 regulates mitochondrial fatty-acid oxidation by reversible enzyme deacetylation. Nature (2010) 464:121–5. 10.1038/nature08778 20203611 PMC2841477

[96] Ho L Titus AS Banerjee KK George S Lin W Deota S. SIRT4 regulates ATP homeostasis and mediates a retrograde signaling via AMPK. Aging (2013) 5:835–49. 10.18632/aging.100616 24296486 PMC3868726

[97] Zhou L Wang F Sun R Chen X Zhang M Xu Q. SIRT5 promotes IDH2 desuccinylation and G6PD deglutarylation to enhance cellular antioxidant defense. EMBO Rep. (2016) 17:811–22. 10.15252/embr.201541643 27113762 PMC5278614

[98] Zhong L D'Urso A Toiber D Sebastian C Henry RE Vadysirisack DD. The histone deacetylase Sirt6 regulates glucose homeostasis via Hif1alpha. Cell (2010) 140:280–93. 10.1016/j.cell.2009.12.041 20141841 PMC2821045

[99] Hu CJ Iyer S Sataur A Covello KL Chodosh LA Simon MC. Differential regulation of the transcriptional activities of hypoxia-inducible factor 1 alpha (HIF-1α) and HIF-2α in stem cells. Mol Cell Biol. (2006) 26:3514–26. 10.1128/MCB.26.9.3514-3526.2006 16611993 PMC1447431

[100] Randle PJ Garland PB Hales CN Newsholme EA. The glucose fatty-acid cycle. Its role in insulin sensitivity and the metabolic disturbances of diabetes mellitus. Lancet (1963) 1:785–9. 13990765 10.1016/s0140-6736(63)91500-9

[101] Furler SM Poynten AM Kriketos AD Lowy AJ Ellis BA Maclean EL. Independent influences of central fat and skeletal muscle lipids on insulin sensitivity. Obes Res. (2001) 9:535–43. 10.1038/oby.2001.70 11557834

[102] Feige JN Lagouge M Canto C Strehle A Houten SM Milne JC. Specific SIRT1 activation mimics low energy levels and protects against diet-induced metabolic disorders by enhancing fat oxidation. Cell Metab. (2008) 8:347–58. 10.1016/j.cmet.2008.08.017 19046567

[103] Iwabu M Yamauchi T Okada-Iwabu M Sato K Nakagawa T Funata M. Adiponectin and AdipoR1 regulate PGC-1α and mitochondria by Ca(2+) and AMPK/SIRT1. Nature (2010) 464:1313–9. 10.1038/nature08991 20357764

[104] Arora A Dey CS. SIRT2 negatively regulates insulin resistance in C2C12 skeletal muscle cells. Biochim Biophys Acta (2014) 1842:1372–8. 10.1016/j.bbadis.2014.04.027 24793418

[105] Jing E Emanuelli B Hirschey MD Boucher J Lee KY Lombard D. Sirtuin-3 (Sirt3) regulates skeletal muscle metabolism and insulin signaling via altered mitochondrial oxidation and reactive oxygen species production. Proc Natl Acad Sci USA. (2011) 108:14608–13. 10.1073/pnas.1111308108 21873205 PMC3167496

[106] Jing E O'Neill BT Rardin MJ Kleinridders A Ilkeyeva OR Ussar S. Sirt3 regulates metabolic flexibility of skeletal muscle through reversible enzymatic deacetylation. Diabetes (2013) 62:3404–17. 10.2337/db12-1650 23835326 PMC3781465

[107] Muoio DM Neufer PD. Lipid-induced mitochondrial stress and insulin action in muscle. Cell Metab. (2012) 15:595–605. 10.1016/j.cmet.2012.04.010 22560212 PMC3348508

[108] Lantier L Williams AS Williams IM Yang KK Bracy DP Goelzer M. SIRT3 is crucial for maintaining skeletal muscle insulin action and protects against severe insulin resistance in high-fat-fed mice. Diabetes (2015) 64:3081–92. 10.2337/db14-1810 25948682 PMC4542443

[109] Nasrin N Wu X Fortier E Feng Y Bare OC Chen S. SIRT4 regulates fatty acid oxidation and mitochondrial gene expression in liver and muscle cells. J Biol Chem. (2010) 285:31995–2002. 10.1074/jbc.M110.124164 20685656 PMC2952200

[110] Kelley DE Goodpaster B Wing RR Simoneau JA. Skeletal muscle fatty acid metabolism in association with insulin resistance, obesity, and weight loss. Am J Physiol. (1999) 277:E1130–41. 10600804 10.1152/ajpendo.1999.277.6.E1130

[111] Kelley DE He J Menshikova EV Ritov VB. Dysfunction of mitochondria in human skeletal muscle in type 2 diabetes. Diabetes (2002) 51:2944–50. 10.2337/diabetes.51.10.2944 12351431

[112] Rodgers JT Puigserver P. Fasting-dependent glucose and lipid metabolic response through hepatic sirtuin 1. Proc Natl Acad Sci USA. (2007) 104:12861–6. 10.1073/pnas.0702509104 17646659 PMC1937557

[113] Purushotham A Schug TT Xu Q Surapureddi S Guo X Li X. Hepatocyte-specific deletion of SIRT1 alters fatty acid metabolism and results in hepatic steatosis and inflammation. Cell Metab. (2009) 9:327–38. 10.1016/j.cmet.2009.02.006 19356714 PMC2668535

[114] Sugden MC Caton PW Holness MJ. PPAR control: it's SIRTainly as easy as PGC. J Endocrinol. (2010) 204:93–104. 10.1677/JOE-09-0359 19770177

[115] Ponugoti B Kim DH Xiao Z Smith Z Miao J Zang M. SIRT1 deacetylates and inhibits SREBP-1C activity in regulation of hepatic lipid metabolism. J Biol Chem. (2010) 285:33959–70. 10.1074/jbc.M110.122978 20817729 PMC2962496

[116] Krishnan J Danzer C Simka T Ukropec J Walter KM Kumpf S. Dietary obesity-associated Hif1alpha activation in adipocytes restricts fatty acid oxidation and energy expenditure via suppression of the Sirt2-NAD+ system. Genes Dev. (2012) 26:259–70. 10.1101/gad.180406.111 22302938 PMC3278893

[117] Lombard DB Alt FW Cheng HL Bunkenborg J Streeper RS Mostoslavsky R. Mammalian Sir2 homolog SIRT3 regulates global mitochondrial lysine acetylation. Mol Cell Biol. (2007) 27:8807–14. 10.1128/MCB.01636-07 17923681 PMC2169418

[118] Hallows WC Yu W Smith BC Devries MK Ellinger JJ Someya S. Sirt3 promotes the urea cycle and fatty acid oxidation during dietary restriction. Mol Cell (2011) 41:139–49. 10.1016/j.molcel.2011.01.002 21255725 PMC3101115

[119] Shimazu T Hirschey MD Hua L Dittenhafer-Reed KE Schwer B Lombard DB. SIRT3 deacetylates mitochondrial 3-hydroxy-3-methylglutaryl CoA synthase 2 and regulates ketone body production. Cell Metab. (2010) 12:654–61. 10.1016/j.cmet.2010.11.003 21109197 PMC3310379

[120] Hirschey MD Shimazu T Jing E Grueter CA Collins AM Aouizerat B. SIRT3 deficiency and mitochondrial protein hyperacetylation accelerate the development of the metabolic syndrome. Mol Cell (2011) 44:177–90. 10.1016/j.molcel.2011.07.019 21856199 PMC3563434

[121] Yoshizawa T Karim MF Sato Y Senokuchi T Miyata K Fukuda T. SIRT7 controls hepatic lipid metabolism by regulating the ubiquitin-proteasome pathway. Cell Metab. (2014) 19:712–21. 10.1016/j.cmet.2014.03.006 24703702

[122] Karim MF Yoshizawa T Sobuz SU Sato Y Yamagata K. Sirtuin 7-dependent deacetylation of DDB1 regulates the expression of nuclear receptor TR4. Biochem Biophys Res Commun. (2017) 490:423–428. 10.1016/j.bbrc.2017.06.057 28623141

[123] Ryu D Jo YS Lo Sasso G Stein S Zhang H Perino A. A SIRT7-dependent acetylation switch of GABPbeta1 controls mitochondrial function. Cell Metab. (2014) 20:856–69. 10.1016/j.cmet.2014.08.001 25200183

[124] Shin J He M Liu Y Paredes S Villanova L Brown K. SIRT7 represses Myc activity to suppress ER stress and prevent fatty liver disease. Cell Rep. (2013) 5:654–65. 10.1016/j.celrep.2013.10.007 24210820 PMC3888240

[125] Laurent G de Boer VC Finley LW Sweeney M Lu H Schug TT. SIRT4 represses peroxisome proliferator-activated receptor alpha activity to suppress hepatic fat oxidation. Mol Cell Biol. (2013) 33:4552–61. 10.1128/MCB.00087-13 24043310 PMC3838178

[126] Rajala MW Scherer PE. Minireview: the adipocyte–at the crossroads of energy homeostasis, inflammation, and atherosclerosis. Endocrinology (2003) 144:3765–73. 10.1210/en.2003-0580 12933646

[127] Berg AH Scherer PE. Adipose tissue, inflammation, and cardiovascular disease. Circ Res. (2005) 96:939–49. 10.1161/01.RES.0000163635.62927.34 15890981

[128] Kadowaki T Yamauchi T. Adiponectin and adiponectin receptors. Endocr Rev. (2005) 26:439–51. 10.1210/er.2005-0005 15897298

[129] Picard F Kurtev M Chung N Topark-Ngarm A Senawong T Machado De Oliveira R. Sirt1 promotes fat mobilization in white adipocytes by repressing PPAR-gamma. Nature (2004) 429:771–6. 10.1038/nature02583 15175761 PMC2820247

[130] Qiang L Wang L Kon N Zhao W Lee S Zhang Y. Brown remodeling of white adipose tissue by SirT1-dependent deacetylation of Ppargamma. Cell (2012) 150:620–32. 10.1016/j.cell.2012.06.027 22863012 PMC3413172

[131] Kurylowicz A Owczarz M Polosak J Jonas MI Lisik W Jonas M SIRT1 and SIRT7 expression in adipose tissues of obese and normal-weight individuals is regulated by microRNAs but not by methylation status. Int J Obes. (2016) 40:1635–42. 10.1038/ijo.2016.131 27480132

[132] Cioffi M Vallespinos-Serrano M Trabulo SM Fernandez-Marcos PJ Firment AN Vazquez BN. MiR-93 controls adiposity via inhibition of Sirt7 and Tbx3. Cell Rep. (2015) 12:1594–605. 10.1016/j.celrep.2015.08.006 26321631

[133] Tong Z Wang Y Zhang X Kim DD Sadhukhan S Hao Q. SIRT7 Is activated by DNA and deacetylates histone H3 in the chromatin context. ACS Chem Biol. (2016) 11:742–7. 10.1021/acschembio.5b01084 26907567 PMC4850736

[134] Tong Z Wang M Wang Y Kim DD Grenier JK Cao J. SIRT7 is an RNA-activated protein lysine deacylase. ACS Chem Biol. (2017) 12:300–10. 10.1021/acschembio.6b00954 27997115 PMC5326686

[135] Kuang J Zhang Y Liu Q Shen J Pu S Cheng S. Fat-specific Sirt6 ablation sensitizes mice to high-fat diet-induced obesity and insulin resistance by inhibiting lipolysis. Diabetes (2017) 66:1159–71. 10.2337/db16-1225 28250020

[136] Ramadori G Fujikawa T Fukuda M Anderson J Morgan DA Mostoslavsky R. SIRT1 deacetylase in POMC neurons is required for homeostatic defenses against diet-induced obesity. Cell Metab. (2010) 12:78–87. 10.1016/j.cmet.2010.05.010 20620997 PMC2904327

[137] Ramadori G Fujikawa T Anderson J Berglund ED Frazao R Michan S. SIRT1 deacetylase in SF1 neurons protects against metabolic imbalance. Cell Metab. (2011) 14:301–12. 10.1016/j.cmet.2011.06.014 21907137 PMC3172583

[138] Sasaki T Kim HJ Kobayashi M Kitamura YI Yokota-Hashimoto H Shiuchi T. Induction of hypothalamic Sirt1 leads to cessation of feeding via agouti-related peptide. Endocrinology (2010) 151:2556–66. 10.1210/en.2009-1319 20375183

[139] Velasquez DA Martinez G Romero A Vazquez MJ Boit KD Dopeso-Reyes IG. The central Sirtuin 1/p53 pathway is essential for the orexigenic action of ghrelin. Diabetes (2011) 60:1177–85. 10.2337/db10-0802 21386086 PMC3064091

[140] Lu M Sarruf DA Li P Osborn O Sanchez-Alavez M Talukdar S. Neuronal Sirt1 deficiency increases insulin sensitivity in both brain and peripheral tissues. J Biol Chem. (2013) 288:10722–35. 10.1074/jbc.M112.443606 23457303 PMC3624452

[141] Roy Chowdhury S Djordjevic J Thomson E Smith DR Albensi BC Fernyhough P Depressed mitochondrial function and electron transport complex II-mediated H2O2 production in the cortex of type 1 diabetic rodents. Mol Cell Neurosci. (2018) 90:49–59. 10.1016/j.mcn.2018.05.006 29802939

[142] Dong Y Guo T Traurig M Mason CC Kobes S Perez J. SIRT1 is associated with a decrease in acute insulin secretion and a sex specific increase in risk for type 2 diabetes in Pima Indians. Mol Genet Metab. (2011) 104:661–5. 10.1016/j.ymgme.2011.08.001 21871827 PMC3224181

[143] Maeda S Koya D Araki SI Babazono T Umezono T Toyoda M. Association between single nucleotide polymorphisms within genes encoding sirtuin families and diabetic nephropathy in Japanese subjects with type 2 diabetes. Clin Exp Nephrol. (2011) 15:381–90. 10.1007/s10157-011-0418-0 21331741 PMC3110272

[144] Botden IP Zillikens MC de Rooij SR Langendonk JG Danser AH Sijbrands EJ. Variants in the SIRT1 gene may affect diabetes risk in interaction with prenatal exposure to famine. Diabetes Care (2012) 35:424–6. 10.2337/dc11-1203 22228742 PMC3263901

[145] Sosnowska B Mazidi M Penson P Gluba-Brzozka A Rysz J Banach M The sirtuin family members SIRT1, SIRT3 and SIRT6: their role in vascular biology and atherogenesis. Atherosclerosis (2017) 265:275–82. 10.1016/j.atherosclerosis.2017.08.027 28870631

[146] Tan MH Alquraini H Mizokami-Stout K MacEachern M. Metformin: from research to clinical practice. Endocrinol Metab Clin North Am. (2016) 45:819–43. 10.1016/j.ecl.2016.06.008 27823607

[147] Sliwinska A Drzewoski J. Molecular action of metformin in hepatocytes: an updated insight. Curr Diabetes Rev. (2015) 11:175–81. 10.2174/1573399811666150325233108 25808533

[148] Knowler WC Barrett-Connor E Fowler SE Hamman RF Lachin JM Walker EA Reduction in the incidence of type 2 diabetes with lifestyle intervention or metformin. N Engl J Med. (2002) 346:393–403. 10.1056/NEJMoa012512 11832527 PMC1370926

[149] Moin T Schmittdiel JA Flory JH Yeh J Karter AJ Kruge LE. Review of metformin use for type 2 diabetes prevention. Am J Prev Med. (2018) 55:565–74. 10.1016/j.amepre.2018.04.038 30126667 PMC6613947

[150] Kaleida, Health,. Effect of Resveratrol on Insulin Resistance and Inflammatory Mediators in Obese and Type 2 Diabetic Subjects, 2010. ClinicalTrials.gov. Identifier: NCT01158417 Available online at: http://clinicaltrials.gov/ct2/show/NCT01158417?term=NCT01158417.&rank = 1

[151] Mellini P Valente S Mai A. Sirtuin modulators: an updated patent review (2012–2014). Expert Opin Ther Pat. (2015) 25:5–15. 10.1517/13543776.2014.982532 25435179

[152] Boocock DJ Faust GE Patel KR Schinas AM Brown VA Ducharme MP. Phase I dose escalation pharmacokinetic study in healthy volunteers of resveratrol, a potential cancer chemopreventive agent. Cancer Epidemiol Biomarkers Prev. (2007) 16:1246–52. 10.1158/1055-9965.EPI-07-0022 17548692

[153] GlaxoSmithKline A Clinical Study to Assess the Safety, Tolerability, and Activity of Oral SRT2104 Capsules Administered for 28 Days to Subjects With Type 2 Diabetes Mellitus. 2011. ClinicalTrials.gov. Identifier: NCT01018017 Available online at: http://clinicaltrials.gov/ct2/show/study/NCT01018017?term=NCT01018017.&rank=1

[154] GlaxoSmithKline A Phase I Randomized, Placebo-Controlled, Single-Blind, Multiple-Dose, Dose-Escalation Clinical Study to Assess the Safety and Pharmacokinetics of SRT2379 in Normal Healthy Male Volunteers, 2011. ClinicalTrials.gov.Identifier: NCT01018628 Available online at: http://clinicaltrials.gov/ct2/show/NCT01018628?term=NCT01018628&rank=1

[155] Rardin MJ He W Nishida Y Newman JC Carrico C Danielson SR. SIRT5 regulates the mitochondrial lysine succinylome and metabolic networks. Cell Metab. (2013) 18:920–33. 10.1016/j.cmet.2013.11.013 24315375 PMC4105152

[156] Cohen HY Miller C Bitterman KJ Wall NR Hekking B Kessler B. Calorie restriction promotes mammalian cell survival by inducing the SIRT1 deacetylase. Science (2004) 305:390–2. 10.1126/science.1099196 15205477

[157] Mathias RA Greco TM Oberstein A Budayeva HG Chakrabarti R Rowland EA. Sirtuin 4 is a lipoamidase regulating pyruvate dehydrogenase complex activity. Cell (2014) 159:1615–25. 10.1016/j.cell.2014.11.046 25525879 PMC4344121

[158] Park J Chen Y Tishkoff DX Peng C Tan M Dai L. SIRT5-mediated lysine desuccinylation impacts diverse metabolic pathways. Mol Cell (2013) 50:919–30. 10.1016/j.molcel.2013.06.001 23806337 PMC3769971

[159] Burns J Yokota T Ashihara H Lean ME Crozier A. Plant foods and herbal sources of resveratrol. J Agric Food Chem. (2002) 50:3337–40. 10.1021/jf0112973 12010007

[160] Hung CH Chan SH Chu PM Tsai KL. Quercetin is a potent anti-atherosclerotic compound by activation of SIRT1 signaling under oxLDL stimulation. Mol Nutr Food Res. (2015) 59:1905–17. 10.1002/mnfr.201500144 26202455

[161] Affuso F Mercurio V Fazio V Fazio S. Cardiovascular and metabolic effects of Berberine. World J Cardiol. (2010) 2:71–7. 10.4330/wjc.v2.i4.71 21160701 PMC2999047

